# Beyond technology usefulness: Health system reliability, and socio-demographic and professional factors as shapers of technostress in physicians

**DOI:** 10.1371/journal.pdig.0001541

**Published:** 2026-07-30

**Authors:** Pilar Ficapal-Cusí, Joan Torrent-Sellens, Oriol Yuguero Torres

**Affiliations:** 1 Faculty of Economics and Business Studies, i2TIC-IA Lab and UOC-DIGIT, Barcelona, Spain; 2 Institut de Recerca Biomédica de Lleida (UdL-IRBLLEIDA), Lleida, Spain; 3 E-RLab and eHealth Centre, Open University of Catalunya (UOC), Barcelona, Spain; University of Pittsburgh School of Medicine, UNITED STATES OF AMERICA

## Abstract

Healthcare digitalisation may improve care yet can undermine clinicians’ wellbeing through technostress. We conducted a cross-sectional online survey of physicians in Catalonia, Spain (March–April 2024; n = 423) using the Technostress Creators Scale to quantify overall technostress and five techno-stressors (overload, invasion, complexity, insecurity and uncertainty). Hierarchical regression with bootstrapped standard errors assessed incremental contributions of demographics, digital experience and use, and contextual factors. Overall technostress was moderate (2.91 ± 0.59/5); techno-overload (3.37 ± 0.82) and techno-uncertainty (3.29 ± 0.74) were highest, while techno-insecurity was lowest (2.15 ± 0.74). Across models, greater professional experience and the interaction between gender (women), hospital care, and location in non-metropolitan areas were significant predictors. A higher probability was also detected in tasks where technology is more prevalent in healthcare, patients also have access to it and are pressured to use it, and where technical problems are frequent. Finally, moderators related to the digital maturity of professionals and better access to, and usability of digital tools were identified. These findings should be interpreted as exploratory associations, given the non-probabilistic convenience sample and the cross-sectional design, which preclude claims about population representativeness, response rate estimation, or causal inference. Nevertheless, the results suggest that promoting physicians’ digital wellbeing requires attention to sociodemographic, professional, and technological factors associated with higher technostress.

## Author summary

Digital technologies are now part of everyday healthcare, but their implementation can also create new sources of stress for clinicians. This study examines technostress among physicians in Catalonia, Spain, focusing not only on the usefulness of digital tools, but also on how reliable, accessible and integrated these tools are in clinical practice. Using an online survey of 423 physicians, we found that overall technostress was moderate, with the highest levels related to technology-driven workload and uncertainty caused by continuous digital change. Older physicians, women working in hospital settings, and professionals working outside metropolitan areas showed higher levels of technostress in some dimensions. Technostress was also higher when physicians used more digital tools in patient care, when patients requested digital interaction, and when digital applications presented technical problems. Conversely, previous experience with telemedicine and eHealth, easier-to-use tools, and better access for citizens were associated with lower technostress. These findings suggest that preventing technostress requires more than training individual professionals: healthcare organisations must improve usability, technical reliability, workflow integration and equitable support for different professional groups.

## Background

The digital transformation of healthcare systems represents one of the most profound changes in the history of medicine [1]. It is reshaping the organisation of care, clinical decision-making, and professional roles to such an extent that future analyses will likely consider it a defining element in the evolution of modern healthcare. Around these changes, new concepts such as **cyber-bioethics** have emerged [2], calling for reflection on the ethical, social, and professional implications of the rapid implementation of digital tools.

However, this accelerated transformation—driven by the massive management of health data, the widespread use of mobile applications, and the integration of artificial intelligence—has also revealed a reality that requires urgent attention: **digital vulnerability**. Individuals already at risk of social or health disadvantage may become even more vulnerable due to the widening **digital divide**, a phenomenon that affects both patients [3] (through the so-called *digital determinants of health*) and healthcare professionals [4].

Among professionals, despite institutional efforts to promote digital competencies, the concept of **technostress** has gained prominence. The term was introduced when technology began to permeate daily life and was originally defined as *“a modern disease of adaptation caused by an inability to cope with new computer technologies in a healthy manner*. Over time, the conceptualisation of TS as an adaptation problem related to difficulties in coping with the physical, social, and cognitive demands of ICT has been consolidated [5,6]. In healthcare, technostress has been linked to decreased job satisfaction, reduced efficiency, and, importantly, to the development of **burnout**, particularly in professional groups that traditionally exhibited low levels of emotional exhaustion [7].

Some recent empirical work suggests that around **40%** of healthcare practitioners may experience medium-to-high levels of technostress (e.g., ~ 41% in a hospital-based sample). Moreover, technostress has been linked to adverse psychological outcomes such as anxiety, depression, mental fatigue, and reduced job performance [8]. While direct evidence on technostress leading to patient safety incidents is limited, concerns have been raised in telehealth literature about how suboptimal digital interactions might compromise care quality and safety [9].

Previous research suggests that the degree of technostress varies according to gender, digital literacy, years of experience, and professional category [10–12]. On the other hand, technostress is reportedly caused by employees’ interaction with digital technologies such as smartphones, laptops, email, and social collaboration software. Several studies indicate that specific features of these tools - particularly their usability and the extent to which they are dynamic or intrusive- are strongly associated with the development of technostress and related techno-stressors [13]Yet, evidence regarding the sociodemographic and organisational factors associated with technostress remains inconsistent. For example, a recent study from the University of Tehran found no clear pattern linking technostress to specific demographic characteristics, highlighting the need for more nuanced analyses [14]. Indeed, the exploration of predictors and mediators of technostress has become an emerging research field over the past few years [15,16].

Given the potential impact of technostress on workforce wellbeing, productivity, and the sustainability of health systems, it is essential to develop **predictive models** that can identify vulnerable professional profiles.

Therefore, this study aims to describe levels of technostress (TS) and its dimensions among physicians, and to **identify and predict the factors associated with overall technostress and each of its dimensions**, in order to better understand differential patterns of vulnerability and adaptation in the context of healthcare digital transformation. This study adopts an exploratory and explanatory approach, providing insight into the factors related to TS and highlighting areas where preventive strategies and organisational support may be needed. The research questions were:

- Is there a significant correlation between sociodemographic characteristics, technology access and technology use, and the levels of technostress (both general and specific factors) in physicians?
- Which sociodemographic characteristics, technology access and technology usage best predict the general level of technostress in physicians?
- Furthermore, which of these predictor variables best predict the variance within each specific technostressor factor (techno-overload, techno-invasion, techno-complexity, techno-insecurity, techno-uncertainty) in physicians?

## Methods

A cross-sectional online survey was conducted among physicians in Catalonia, Spain, to assess the prevalence of the TS, examine its dimensions, and determine their association with sociodemographic characteristics and the patterns of technology use. This research adopts an explanatory study design with exploratory correlational analysis. Spearman’s rank correlation (ρ) was first used to explore associations among variables, followed by ordinary least squares and discrete choice regression models to explain the contribution of predictor blocks to the TS and technostressors.

### Research participants and procedure

Data were collected cross-sectionally from a convenience sample of physicians from primary care and hospital settings employed in the health sector of Catalonia. An online self-reported survey Qualtric platform was used, previously proposed and validated questionnaires.

The questionnaire was conducted between 1 March and 30 April 2024. It required approximately 10 min to complete. It was sent to all professionals forming the study universe. Dissemination of the survey was conducted across the official channels of professional networks, including regional medical associations, and via open calls posted on social media platforms. We estimate that the survey was potentially accessible to over 2000 physicians within the health sector of Catalonia. However, due to this dissemination approach, it was unfeasible to determine the exact number of physicians who received or accessed the invitation to the questionnaire. The inability to calculate a precise response rate necessitates that the representativeness of the sample be interpreted with appropriate caution. We submit that this design is nevertheless appropriate for the inferential aims of the research [17].

We collected 512 responses and 423 were complete. The inclusion criterion required that all survey items be fully answered, incomplete or inconsistent responses were excluded. The sample characteristics are shown in Table 1.

**Table 1:** Frequencies of sociodemographic, work-related characteristics and uses of DT among physicians (n = 423).

Participants	Freq.	Perc.
**Gender**		
Female	267	63.1
Male	156	36.9
**Age**		
21-30	10	2.4
31-40	71	16.8
41-50	103	24.3
51-60	132	31.2
> 60	107	25.3
**Work setting**		
1. Primary care	131	31.0
2. Hospital care	230	54.4
3. Social and health care	3	0.7
4. Other	59	13.9
**Years in practice**		
< 1	5	1.2
1-5	22	5.2
6-10	48	11.3
11-20	96	22.7
> 20	252	59.6
**Tenure in the healthcare centre**		
< 1	22	5.2
1-5	93	22.0
6-10	64	15.1
11-20	102	24.1
> 20	142	33.6
**General uses of DT**		
No use	2	0.5
Management and clinical practice	338	79.9
General in relation to the patient	69	16.3
General in research	14	3.3
**Use in daily clinical practice**		
Management of appointments and referrals	307	72.6
Management of temporary disabilities	108	25.5
Prescription of medications	311	73.5
Requests and results of tests	353	83.5
Clinical problems	283	66.9
Clinical follow-ups	317	74.9
Reminders	199	47.0
Advice and user inquiries	164	38.8
Sending information	229	54.1
**Experience**		
I am a habitual user of social media and digital platforms (both professionally and personally)	255	60.3
I have previous experience using telemedicine systems and digital health	167	39.5
**Intention to use and usefulness**		
I feel that digital health tools and platforms for my tasks are very easy to use.	172	40.7
I feel that digital tools are useful for managing patient scheduling and optimizing visit appropriateness	279	66.0
Digital technologies are very useful for the career development	332	78.5
		
**Contextual influences**		
My colleagues use digital health tools and applications often	249	58.9
The service that I work on encourages and facilitates the use of digital health tools and apps	233	55.1
Some of my patients ask me to use it	141	33.3
Care professionals can access digital health tools and applications very easily	208	49.2
Citizens can access digital health tools and apps very easily	195	46.1
Digital health tools and applications present technical issues	278	65.7

No formal a priori sample size calculation was performed due the absence of a defined sampling frame. However, the final sample of 423 physicians satisfies standard methodological criteria for cross-sectional studies. According to Green’s rule of thumb for regression analysis (N ≥ 50 + 8 m) our sample size provides sufficient power to examine associations with multiple final predictors and the minimum requirements to ensure the stability of the regression coefficients. Additionally, the achieved sample size (N = 423) yields a case-to-predictor ratio of 42:1 for the ten-parameter specification (reported in Table 4), comfortably above the 10:1–20:1 thresholds conventionally recommended for OLS inference [18]

## Measures

### Technostress creators scale

TS was measured using the Spanish version of the Technostress Creators Scale (TCS), adapted and validated into Spanish and used in other studies [19]. The TCS is a 23-item instrument that assesses five dimensions of Technostress (TS) or technostressors: techno-overload (TO): technology-driven excess workload (five items); techno-invasion (TI): work–life interference (four items); techno-complexity (TC): perceived difficulty in managing digital technologies (five items); techno-insecurity (TIN): fear of job displacement by technological systems (five items); and techno-uncertainty (TU): uncertainty driven by continual technology updates (4 items).

Participants scored the items on a Likert scale from 1 to 5, with 1 being ‘strongly disagree’ and 5 ‘strongly agree’. The overall TS level was calculated as the arithmetic mean of the scores across all five dimensions. The internal consistency of the scale was assessed using Cronbach’s alpha (α). In this study, the overall reliability coefficient was 0.90, indicating strong internal consistency. Similarly, the internal consistency of each subscale demonstrated good reliability, with all α values exceeding the recommended threshold of 0.70 [20]. Specifically, the reliability coefficients were: TO=0.80; TI = 0.82; TC = 0.89; TIN = 0.85; and TU = 0.75.

### Demographic and work-related characteristics

Survey items captured participants’ demographic and work-related characteristics (gender, age, professional profile, work setting, years of practice, and tenure in the healthcare centre).

### Digital technologies: experience, usages and context influences

Consistent with the Transactional Model of Stress (TMS), the study included variables related to domain-especific technology use, intention of use and perceived usefullness of digital technologies, as well as usage experience and contextual factors [21,22].

The use of digital technologies in daily clinical practice was assessed across nine core professional activities (e.g., *I use digital technologies to send information*) using a 5-point Likert scale (1 = very little use, 5 = very high use). Additionally, an 11-item instrument assessed three domains: experience with DT (e.g., *Previous experience using telemedicine systems and digital health tools*); intention of use and perceived usefulness of digital technologies (e.g., *Digital technologies are useful for career development*); and contextual influences (e.g., *Colleagues frequently use digital health tools and applications*) [23]. All items were rated on a 5-point Likert scale ranging from 1 (strongly disagree) to 5 (strongly agree). The instrument demonstrated good internal consistency (α = 0.77), indicating acceptable reliability.

### Representativeness and common method bias

This study relies on an incidental sample of 423 practising physicians in Catalonia recruited through professional channels (medical-college mailings, professional-society networks and social-media dissemination), for which a conventional response rate cannot be computed because the exposure denominator is undefined. This design precludes claims about population prevalence but does not compromise the multivariate associations that are the inferential focus of the manuscript [24], and is consistent with the dominant practice in the empirical literature on technostress in healthcare professionals [25]. Common method bias was assessed using Harman’s single-factor test [26]. This procedure was conducted through Principal Component Analysis (PCA) with an unrotated solution to examine whether a single factor dominated the variance structure. The analysis showed that the first factor accounted for 16.9% of the total variance, well below the commonly accepted 50% threshold. These results indicate that CMB is unlikely to be present in this study.

### Data analysis

Descriptive statistics were used to assess the prevalence of TS and each technostressor (TO, TI, TC, TIN, and TU) among physicians.

Prior to the analysis, normality was assessed using the Kolmogorov–Smirnov test, skewness and kurtosis values, and inspection of histograms. Given the non-normal distribution of the data, non-parametric correlation analyses were conducted. Specifically, Spearman correlation coefficient (ρ) was computed to assess the association between the variables [27]. The examination of the correlation matrix made it possible to identify associations between variables and to explore potential multicollinearity prior to running the regression analysis.

We follow the usual analytical strategy for censored health-status measures [28], and test and report three complementary Ordinary Least Squares (OLS) and Tobit regression estimators under floor and ceiling effects [29]. OLS is the dominant estimator in the empirical literature on technostress in healthcare settings [30]. Under classical assumptions, OLS coefficients are unbiased and efficient when the dependent variable is continuous and unbounded. We use OLS as the reference model because it remains the most parsimonious and interpretable specification when censoring estimated associations are weak (as is the case in our data) and because it ensures comparability with prior empirical work on physicians’ technostress [31].

The OLS specification has been contrasted with two additional regression models [32]. The doubly censored Tobit model treats the observed outcome as the realization of a latent continuous variable. Its maximum likelihood estimation accounts for the probability mass that accumulates at either bound, yielding consistent coefficients when an OLS regression on the same data would be biased. The Tobit specification is the natural primary model when censoring is not negligible, and a robustness test for the OLS model otherwise.

Tobit’s β coefficients refer to the latent variable and are not directly comparable to OLS coefficients, which estimate the effects on a directly observed variable. To recover an interpretation comparable to OLS, while maintaining censorship correction, we have estimated the average marginal effects (AME) on the conditional expected value of the observed outcome. The AME reduces the latent β to the OLS coefficient through this probability, establishing a link between the OLS estimate (which ignores censorship) and the Tobit β estimate (which lies on the latent scale). Thus, the AME preserves Tobit’s censorship correction while sharing the observed scale of OLS and is therefore the natural quantity for any quantification of policies or interventions [33].

In summary, triangulation between OLS, Tobit, and OLS-Tobit (AME) allows us to verify whether qualitative inferences are robust to the choice of estimator and to detect dimensions where the choice of estimator materially affects the magnitudes.

### Inclusion and ethics statement

The study was conducted anonymously, and required informed consent for participation. Participation was entirely voluntary, and no form of compensation or gratification was provided to respondents. All participants were explicitly informed of the security and privacy protocols applied to the data they provided. The study adhered to the recommendations of the Declaration of Helsinki. Data management was conducted in compliance with Spain’s Organic Law 3/2018 on Personal Data Protection and Guarantee of Digital Rights (LOPD-GDD 3/2018) and the European Union’s General Data Protection Regulation (GDPR, Regulation 2016/679).

### Patient and public involvement

Patients or the public were not involved in the design, or conduct, or reporting, or dissemination plans of our research

## Results

### Sample characteristics

The study sample comprised 423 physicians. Most respondents were women (63.1%) and over 40 years of age (80.8%), with a mean age of 51.3 years. The sample showed a high level of professional experience, as 82.3% of participants reported more than 10 years of work experience, and 57.7% had been employed at their current care centre for over a decade. Regarding the care setting, 54.4% of participants worked in hospital care, while 31.0% were employed in primary care. Physicians’use of digital technologies is detailed in Table 1.

When examining the general construct of TS, the data revealed a moderate arithmetic mean score of 2.91 ± 0.59 (mean±SD) (observed range = 1.00–4.7; theoretical range = 1–5). Among the technostressors, techno-overload (TO) (3.37 ± 0.82) and techno-uncertainty (TU) (3.29 ± 0.74) showed the highest mean scores; whereas techno-insecurity (TIN) presented the lowest mean value (2.15 ± 0.74). Scores for techno-invasion (TI) and techno-complexity (TC) were intermediate (see Table 2).

**Table 2:** Prevalence of TS and its dimensions among physicians.

	x ± SD	Min	Max
TCS	2.91 ± 0.59	1,00	4,72
TO	3.37 ± 0.82	1,00	5,00
TI	2.71 ± 0.96	1,00	5,00
TC	3.03 ± 0.93	1,00	5,00
TIN	2.15 ± 0.74	1,00	5,00
TU	3.29 ± 0.74	1,00	5,00

Note: Abbreviation: TS = technostress; TO = techno-overload; TI = techno-invasion; TC = techno-complexity; TIN = techno-insecurity; TU = techno-uncertainty; x = mean; SD = Standard deviation.

### Association between physicians’ characteristics, digital technologies, contextual factors, and technostress

Spearman correlation coefficient (ρ) was estimated to determine the associations among the variables. Table 3 presents the bivariate associations between the overall TS and five dimensions -techno-overload (TO), techno-invasion (TI), techno-complexity (TC), techno-insecurity (TIN) and techno-uncertainty (TU)- and sociodemographic and work-related variables and digital-use variables. Spearman’s rank-order correlations matrix among the study variables are presented in Table 3, including correlation coefficients (ρ) and associated p-values.

**Table 3:** Spearman’s rank-order correlations among the study variables.

	TS		TO		TI		TC		TIN		TU	
**Variables**	**ρ**	**p-value**	**ρ**	**p-value**	**ρ**	**p-value**	**ρ**	**p-value**	**ρ**	**p-value**	**ρ**	**p-value**
Gender^+^	-0.001	0.979	-0.008	0.862	0.019	0.695	0.001	0.976	0.015	0.764	-0.048	0.321
Age	0.232**	**0.000**	0.112*	**0.021**	0.017	0.726	0.303**	**0.000**	0.236**	**0.000**	0.195**	**0.000**
Primary care	0.022	0.655	0.117*	**0.016**	-0.042	0.394	0.036	0.464	-0.061	0.212	0.022	0.652
Hospital care	-0.096*	**0.048**	-0.126**	**0.009**	-0.014	0.770	-0.108*	**0.026**	-0.017	0.723	-0.050	0.301
Social and health care	0.073	0.131	0.038	0.432	0.030	0.543	0.078	0.108	0.076	0.117	0.025	0.612
Other	0.092	0.060	0.017	0.729	0.069	0.158	0.089	0.066	0.087	0.072	0.037	0.446
Years in practice	0.171**	**0.000**	0.099*	**0.041**	-0.002	0.959	0.211**	**0.000**	0.161**	**0.001**	0.180**	**0.000**
Tenure in the healthcare centre	0.180**	**0.000**	0.061	0.214	-0.022	0.651	0.291**	**0.000**	0.178**	**0.000**	0.137**	**0.005**
No use of TD	0.060	0.215	-0.046	0.344	0.024	0.621	0.058	0.236	0.102*	**0.036**	0.078	0.109
Management and clinical practice	-0.029	0.547	-0.002	0.968	0.030	0.542	-0.026	0.587	-0.072	0.139	0.006	0.903
General in relation to the patient	-0.012	0.803	0.001	0.987	-0.072	0.138	-0.004	0.933	0.002	0.961	0.004	0.932
General in research	0.068	0.164	0.020	0.675	0.073	0.132	0.046	0.348	0.117*	**0.016**	-0.052	0.287
Management of appointments and referrals	0.039	0.423	0.185**	**0.000**	0.011	0.819	-0.040	0.410	-0.074	0.129	0.063	0.194
Management of temporary disabilities	0.084	0.083	0.101*	**0.038**	0.057	0.242	0.043	0.379	-0.014	0.781	0.114*	**0.019**
Prescription of medications	-0.059	0.226	-0.020	0.675	-0.075	0.122	-0.089	0.067	-0.094	0.052	0.037	0.443
Requests and results of tests	-0.006	0.900	0.033	0.496	-0.014	0.770	-0.066	0.173	-0.094	0.053	0.098*	**0.044**
Clinical problems	0.005	0.926	0.000	0.993	-0.016	0.745	-0.052	0.282	-0.030	0.532	0.116*	**0.017**
Clinical follow- ups	0.013	0.788	0.053	0.278	-0.018	0.711	-0.060	0.216	-0.054	0.269	0.144**	**0.003**
Reminders	0.102*	**0.036**	0.115*	**0.018**	0.083	0.090	-0.006	0.909	0.059	0.225	0.089	0.067
Advice and user inquiries	0.045	0.359	0.037	0.451	0.081	0.096	-0.020	0.688	0.004	0.938	0.009	0.846
Sending information	0.069	0.155	0.106*	**0.030**	0.070	0.151	-0.060	0.221	0.028	0.571	0.108*	**0.026**
Habitual user of social media and digital platforms	-0.117*	**0.016**	-0.094	0.053	0.014	0.779	-0.160**	**0.001**	-0.112*	**0.021**	-0.109*	**0.025**
Previous experience using telemedicine systems and digital health	-0.123*	**0.011**	-0.117*	**0.016**	0.026	0.590	-0.314**	**0.000**	-0.055	0.257	-0.004	0.935
Digital health tools and platforms used for tasks are very easy to use	-0.269**	**0.000**	-0.278**	**0.000**	-0.158**	**0.001**	-0.304**	**0.000**	-0.111*	**0.023**	-0.118*	**0.015**
Digital tools are useful for managing patient scheduling and optimizing visit appropriateness	-0.091	0.061	-0.129**	**0.008**	-0.047	0.335	-0.100*	**0.039**	0.014	0.776	-0.052	0.289
DT are very useful for the career development	-0.097*	**0.045**	-0.104*	**0.032**	-0.005	0.916	-0.101*	**0.038**	-0.097*	**0.047**	-0.056	0.253
Colleagues use digital health tools and applications often	0.087	0.073	-0.005	0.922	0.061	0.208	0.036	0.464	0.076	0.120	0.143**	**0.003**
Service encourages and facilitates the use of digital health tools and apps	0.077	0.114	0.064	0.192	0.022	0.655	0.016	0.748	0.009	0.852	0.165**	**0.001**
Some of my patients ask me to use it	0.101*	**0.037**	0.037	0.450	0.108*	**0.027**	0.011	0.825	0.071	0.147	0.074	0.129
Care professionals can access digital health tools and applications very easily	-0.203**	**0.000**	-0.178**	**0.000**	-0.138**	**0.004**	-0.241**	**0.000**	-0.120*	**0.014**	-0.050	0.308
Citizens can access digital health tools and apps very easily	-0.183**	**0.000**	-0.165**	**0.001**	-0.103*	**0.034**	-0.172**	**0.000**	-0.135**	**0.005**	-0.104*	**0.032**
Digital health tools and applications present technical issues	0.234**	**0.000**	0.235**	**0.000**	0.096*	**0.049**	0.216**	**0.000**	0.066	0.174	0.197**	**0.000**
												

Notes: TS = technostress; TO = techno-overload; TI = techno-invasion; TC = techno-complexity; TIN = techno-insecurity; TU = techno-uncertainty; ρ = Spearman’s coefficient; p-value: Significant value. ^+^Gender was dummy coded (1 = female, 2 = male).

The strongest associations were observed between age and the technostress dimensions. The strongest associations were observed between age and the TS (ρ = 0.23, p < 0.001) and dimensions TC, TIN, and TU (ρ = 0.19–0.30, p < 0.001), with a weaker association for TO (p < 0.05). Years in practice also showed positive associations with TS (ρ = 0.17, p < 0.001) and dimensions TC, TIN, and TU (ρ = 0.16–0.21, p < 0.001), and a weaker association with TO (ρ = 0.10, p < 0.05).

Most DT use modalities did not show significant relationships with the technostress dimensions assessed. However, specific associations were observed with the TIN dimension. Non-use of DT in the workplace showed a positive and significant correlation with TIN (ρ = 0.10, p < 0.05), indicating that individuals who do not use DT in their work experience greater technological insecurity. General DT use in research also demonstrated a positive and significant correlation with TIN, which may reflect feelings of uncertainty or lack of mastery when using digital tools in research contexts (ρ = 0.12, p < 0.05). Overall, items related to specific uses of DT showed only isolated or moderate associations with the TS and its dimensions, indicating that only certain patterns of technology use are significantly linked to perceived technostress. A significant positive correlation was observed between TO and management of appointments and referrals (ρ = 0.18, p < 0.001) and with management of temporary disabilities (ρ = 0.10, p < 0.05). Likewise, significant correlations were found between TU and the use of technologies for test requests and results and clinical problems (ρ = 0.10–0.12, p < 0.05), as well as for clinical follow-ups (ρ = 0.14, p < 0.01).

Intention to use and perceived usefulness of digital tools showed negative correlations with TS (ρ = –0.10 - –0.27, p < 0.05 to p < 0.001), TC (ρ = –0.10- –0.30, p < 0.05 to p < 0.001) and TIN (ρ = –0.10 - –0.11, p < 0.05) indicating lower stress among professionals perceiving better usability and utility. The experience in telemedicine shows a significant association with TS (ρ = –0.12, p < 0.05); TO (ρ = –0.12, p < 0.05) and TC (ρ = –0.31, p < 0.001), suggesting that greater experience is linked to lower levels of technostress. In the same line, habitual use of social media and digital shows a significant association with TS (ρ = –0.12, p < 0.05); TC (ρ = –0.16, p < 0.001), TU (ρ = –0.11, p < 0.05), and TIN (ρ = –0.11, p < 0.05).

Regarding contextual influences, accessibility to the apps for both citizens and physicians is negatively associated with technostress (ρ = –0.18 - –0.20, p < 0.001) and with most of its dimensions: TO (ρ = –0.17 - –0.18, p < 0.001); TC (ρ = –0.17 - –0.24, p < 0.001); TI (ρ = –0.10 - –0.14, p < 0.05); and TIN (ρ = –0.14 - –0.12, p < 0.05). Moreover, acces to the apps for citizens is negatively associated with TU (ρ = –0.10, p < 0.05).

However, patients’ requests for access to the apps are positively associated with technostress (ρ = –0.10, p < 0.05) and with TI (ρ = –0.11, p < 0.05). Finally, technical issues in digital health tools and applications showed a positive association with TS (ρ = 0.23, p < 0.001) and dimensions: TO (ρ = 0.24, p < 0.001); TC (ρ = 0.22, p < 0.001); TU (ρ = 0.20, p < 0.001); and TI (ρ = 0.10, p < 0.05). When service promote and facilitate the use of DT in healthcare, higher levels of TU are observed (ρ = 0.17, p < 0.001).

### Associated factors to technostress and technostressors

The final specification was selected based on theoretical relevance, previous evidence from studies on technostress in healthcare professionals, and statistical contribution to model fit. The model estimates ten predictors grouped into three blocks. The first block captures the sociodemographic context through physicians’ age, which has been repeatedly identified as a determinant of digital health adoption and technostress in physicians [34]. Additionally, gender and geographical heterogeneity are captured through two interaction terms: women in hospital care and women in metropolitan areas. These terms reflect the systematic gradients of interaction between gender, professional care context, and geographical location in digital health adoption and its associated challenges [35].

A second block measures the use of technology applied to patient interaction (the sum of ICT elements used in the patient relationship domain). This pattern indicates that it is the type and intensity of clinical ICT use, rather than ICT use itself, that drives technostress [9,36]. The third section measures specific dimensions of technology use through five recoded items about user type that capture experience with telemedicine and digital health, perceived ease of use of the tools, patient demand for digital interaction, ease of access to applications for citizens, and the occurrence of technical problems. The selection of these five items as primary predictors of technology use reflects the barriers and facilitators already reported in numerous studies [37,38]

### Overall technostress factors

Table 4 reports the OLS, Tobit and OLS-Tobit (AME) estimates for the factors associated with overall technostress. The OLS specification was first estimated and validated in IBM SPSS Statistics 29 and replicated in Python (stats models) up to the fourth decimal place in every coefficient, standard error and p-value. The model is statistically significant (F(9, 413) = 12.18, p < 0.001) and explains 21.0% of the variance in overall technostress (adjusted R² = 0.193). All ten predictors are statistically significant at conventional levels: nine at p < 0.05, six at p < 0.01, and three at p < 0.001. Censoring is virtually absent in the dependent variable: only one of the 423 observations attains the lower bound of 1 and none reaches the upper bound of 5, so the AME scaling factor equals 0.999 and OLS, Tobit and AME estimates are numerically indistinguishable: a stable empirical pattern when censoring shares are below 5% of the sample. The substantive interpretation of the model is therefore invariant to the choice of estimator. The Tobit specification serves here as a robustness check, confirming that the OLS estimates are not attenuated by boundary effects and can be reported as the primary results without bias concerns.

**Table 4:** Explanatory factors of overall technostress (TS) in physicians.

Variable	OLS	Tobit	OLS-Tobit
	*Coef. (p)*	*Latent β (p)*	*AME (p)*
Constant	2.346*** (<0.001)	2.352*** (<0.001)	2.350*** (<0.001)
Age (years)	0.012*** (<0.001)	0.011*** (<0.001)	0.012*** (<0.001)
Women × Hospital care (interaction)	0.146* (0.015)	0.147* (0.013)	0.147* (0.013)
Women × Metropolitan area (interaction)	-0.144* (0.011)	-0.145** (0.009)	-0.145** (0.009)
Experience with telemedicine and digital health	-0.086** (0.008)	-0.087** (0.007)	-0.087** (0.007)
Some patients request it	0.096** (0.007)	0.095** (0.006)	0.096** (0.006)
Citizens easily access apps	-0.101** (0.003)	-0.102** (0.002)	-0.101** (0.002)
Apps cause technical problems	0.109* (0.016)	0.108* (0.016)	0.108* (0.016)
Tools are easy to use	-0.150*** (<0.001)	-0.149*** (<0.001)	-0.150*** (<0.001)
Sum of ICT use for patient relationship	0.033* (0.018)	0.032* (0.016)	0.033* (0.016)
***Model statistics***			
N (observations)	423	423 (lower cens.: 1; upper cens.: 0)	423
R (multiple correlation)	0.458	—	—
R² (adjusted R²)	0.210 (0.193)	—	—
Log-likelihood	—	-332.24	-332.24
AIC/ BIC	680.6/ 721.0	686.5/ 731.0	686.5/ 731.0
F (p)	12.18 (<0.001)	—	—
σ (scale/ RMSE)	0.528	0.529	0.529
AME scaling factor	—	—	0.999

***Note.*** *Unstandardised coefficients (B); p-values in parentheses. OLS: ordinary least squares estimation on all 423 observations, exactly replicating the SPSS output. Tobit: doubly-censored Tobit regression in* [1, 5] estimated by maximum likelihood; β refers to the latent variable y*. OLS-Tobit: average marginal effects (AME) of the Tobit, ∂E[y|x]/∂x_j_ = β_j_· 1/N· Σ_i_ [Φ((U − X_i_β)/σ̂) − Φ((L − X_i_β)/σ̂)], directly comparable to OLS coefficients. *** p < 0.001; ** p < 0.01; * p < 0.05.

The three estimators yield numerically equivalent coefficients across the entire model. The Tobit β values differ from the OLS coefficients by less than 0.001 in absolute value for every predictor, reflecting the negligible amount of censoring in the dependent variable (1/423 = 0.24%). The AME column lies, as expected, between OLS and Tobit β, but at the same numerical level. The qualitative inferences (significance, sign, and relative ordering of the predictors) are virtually identical across the three estimators. This outcome suggests that overall technostress is an outcome variable that, although bounded, behaves effectively as a continuous variable in the observed range, as literature predicts for situations with very low censoring shares. We therefore report OLS as the primary model and use the Tobit and AME columns as a robustness check.

Regarding coefficients, age is the strongest sociodemographic predictor of overall technostress (b = +0.012, p < 0.001; standardized β = +0.217), indicating that each additional year of age corresponds to roughly 0.012 points of additional technostress on the 1-to-5 scale, or approximately 0.12 points across a decade. This finding is consistent with well-established patterns into eHealth and technostress literature^31^. Older physicians are systematically less likely to endorse efficiency-related facilitators of telemedicine adoption, suggesting a parallel cohort estimated association in technology-related stress responses [34].

The two interaction gender terms are statistically significant and operate in opposite directions. Women working in hospital settings exhibit higher overall technostress than the corresponding reference group (Women × Hospital: b = +0.146, p = 0.015), whereas women working in metropolitan areas generate lower technostress (Women × Metropolitan: b = −0.144, p = 0.011). The two effects are of nearly identical magnitude in absolute terms, indicating that the gendered impact of digitalization on physicians is highly contextual: it intensifies in the hospital and non-metropolitan settings.

Regarding professional settings, the aggregate measure of ICT use applied to patient relationships is positively associated with technostress (b = +0.033, p = 0.018; standardized β = +0.112). Each additional ICT tool used in the patient-interaction domain raises the predicted technostress by approximately 0.033 points on the 1-to-5 scale. This evidence supports literature findings, namely that the volume and breadth of digital-tool use in clinician-patient interaction is a primary structural driver of physician digital stress [38,39]

Regarding technology engagement, five recoded type-of-user items contribute jointly to the explanation of overall technostress, with effects of comparable magnitude but opposite signs that, considered together, produce a coherent and clinically meaningful pattern. Three items act as protective factors: physicians’ experience with telemedicine and digital health (b = −0.086, p = 0.008), perceived ease of use of the tools (b = −0.150, p < 0.001), and citizens’ ease of access to apps (b = −0.101, p = 0.003). Two items act as risk factors: patient demand for digital interaction (b = +0.096, p = 0.007) and the occurrence of technical problems with the apps (b = +0.108, p = 0.017). Several inferences follow from this pattern. First, perceived ease of use is the single most influential predictor in the entire model in standardized terms (β = −0.206), confirming the centrality of usability for digital health professionals’ well-being [32,37]. Second, telemedicine experience is genuinely protective: physicians with greater familiarity and accumulated experience with digital health show measurably lower technostress, after controlling for age, ICT volume and the technology engagement items [30]. Third, patient pull is a stress factor: when patients actively request digital interaction, physicians experience greater technostress, even after controlling for usability and personal experience with the eHealth tools [34]. And fourth, technical problems with the eHealth apps are an independent risk factor whose estimated association is not absorbed by the perceived ease-of-use coefficients [40].

### Technostressors factors

Tables 5–Table 9 present the results of the estimates for the 5 technostressors. The five models present a clear gradient of explanatory power. Techno-complexity is by a substantial margin the best-explained dimension (adjusted R² = 0.266), followed by techno-overload (0.127), techno-insecurity (0.108), techno-uncertainty (0.085), and techno-invasion (0.046). This gradient is theoretically informative: the dimensions that are most directly tied to observable user-tool interactions (complexity and overload) are the most explainable by the objective specification adopted here, while the dimensions that depend on deeper psychosocial mechanisms (invasion, uncertainty) and on broader systemic perceptions (insecurity) are explained more modestly by the same set of predictors. This finding is coherent with the dominant theoretical framework of technostress, in which the five dimensions are postulated to capture distinct stress mechanisms with partially overlapping but not identical determinants [40].

**Table 5:** Explanatory factors of techno-overload (TO) in physicians.

Variable	OLS	Tobit	OLS-Tobit
	*Coef. (p)*	*Latent β (p)*	*AME (p)*
Constant	2.919*** (<0.001)	2.925*** (<0.001)	2.834*** (<0.001)
Age (years)	0.004 (0.226)	0.004 (0.234)	0.004 (0.234)
Women × Hospital care (interaction)	-0.015 (0.865)	-0.016 (0.853)	-0.016 (0.853)
Women × Metropolitan area (interaction)	-0.010 (0.905)	-0.016 (0.844)	-0.016 (0.844)
Experience with telemedicine and digital health	-0.072 (0.120)	-0.073 (0.124)	-0.071 (0.124)
Some patients request it	0.059 (0.241)	0.055 (0.291)	0.053 (0.291)
Citizens easily access apps	-0.088 (0.067)	-0.094 (0.056)	-0.091 (0.056)
Apps cause technical problems	0.213** (0.001)	0.223*** (<0.001)	0.216*** (<0.001)
Tools are easy to use	-0.221*** (<0.001)	-0.232*** (<0.001)	-0.225*** (<0.001)
Sum of ICT use for patient relationship	0.059** (0.003)	0.063** (0.002)	0.062** (0.002)
***Model statistics***			
N (observations)	423	423 (lower cens.: 3; upper cens.: 16)	423
R (multiple correlation)	0.382	—	—
R² (adjusted R²)	0.146 (0.127)	—	—
Log-likelihood	—	-500.83	-500.83
AIC/ BIC	981.8/ 1022.3	1023.7/ 1068.2	1023.7/ 1068.2
F (p)	7.84 (<0.001)	—	—
σ (scale/ RMSE)	0.754	0.785	0.785
AME scaling factor	—	—	0.969

***Note.*** *Unstandardised coefficients (B); p-values in parentheses. OLS: ordinary least squares estimation on all 423 observations, exactly replicating the SPSS output. Tobit: doubly-censored Tobit regression in [*1, 5] estimated by maximum likelihood; β refers to the latent variable y*. OLS-Tobit: average marginal effects (AME) of the Tobit, ∂E[y|x]/∂x_j_ *= β*_j_*· 1/N· Σ*_i_ *[Φ((U − X*_i_*β)/σ̂) −* *Φ((L − X_i_β*)/*σ̂)]*, directly comparable to OLS coefficients. *** p < 0.001; ** p < 0.01; * p < 0.05.

**Table 6:** Explanatory factors of techno-invasion (TI) in physicians.

Variable	OLS	Tobit	OLS-Tobit
	*Coef. (p)*	*Latent β (p)*	*AME (p)*
Constant	2.812*** (<0.001)	2.892*** (<0.001)	2.726*** (<0.001)
Age (years)	-0.001 (0.852)	-0.001 (0.797)	-0.001 (0.797)
Women × Hospital care (interaction)	0.148 (0.160)	0.154 (0.161)	0.145 (0.161)
Women × Metropolitan area (interaction)	-0.125 (0.208)	-0.143 (0.167)	-0.135 (0.167)
Experience with telemedicine and digital health	0.035 (0.538)	0.030 (0.619)	0.028 (0.619)
Some patients request it	0.140* (0.025)	0.141* (0.029)	0.133* (0.029)
Citizens easily access apps	-0.119* (0.044)	-0.128* (0.037)	-0.121* (0.037)
Apps cause technical problems	0.017 (0.831)	0.007 (0.928)	0.007 (0.928)
Tools are easy to use	-0.209*** (<0.001)	-0.217*** (<0.001)	-0.205*** (<0.001)
Sum of ICT use for patient relationship	0.039 (0.109)	0.041 (0.108)	0.038 (0.108)
***Model statistics***			
N (observations)	423	423 (lower cens.: 13; upper cens.: 10)	423
R (multiple correlation)	0.257	—	—
R² (adjusted R²)	0.066 (0.046)	—	—
Log-likelihood	—	-590.44	-590.44
AIC/ BIC	1160.2/ 1200.7	1202.9/ 1247.4	1202.9/ 1247.4
F (p)	3.26 (<0.001)	—	—
σ (scale/ RMSE)	0.931	0.978	0.978
AME scaling factor	—	—	0.943

***Note.*** *Unstandardised coefficients (B); p-values in parentheses. OLS: ordinary least squares estimation on all 423 observations, exactly replicating the SPSS output. Tobit: doubly-censored Tobit regression in* [1, 5] estimated by maximum likelihood; β refers to the latent variable y*. OLS-Tobit: average marginal effects (AME) of the Tobit, ∂E[y|x]/∂x_j_ = β_j_· 1/N· Σ_i_ [Φ((U − X_i_β)/σ̂) − Φ((L − X_i_β)/σ̂)], directly comparable to OLS coefficients. *** p < 0.001; ** p < 0.01; * p < 0.05.

**Table 7:** Explanatory factors of techno-complexity (TC) in physicians.

Variable	OLS	Tobit	OLS-Tobit
	*Coef. (p)*	*Latent β (p)*	*AME (p)*
Constant	2.258*** (<0.001)	2.239*** (<0.001)	2.147*** (<0.001)
Age (years)	0.025*** (<0.001)	0.027*** (<0.001)	0.025*** (<0.001)
Women × Hospital care (interaction)	0.144 (0.107)	0.160 (0.087)	0.153 (0.087)
Women × Metropolitan area (interaction)	-0.237** (0.005)	-0.244** (0.006)	-0.234** (0.006)
Experience with telemedicine and digital health	-0.309*** (<0.001)	-0.323*** (<0.001)	-0.309*** (<0.001)
Some patients request it	0.132* (0.012)	0.134* (0.015)	0.128* (0.015)
Citizens easily access apps	-0.094 (0.061)	-0.096 (0.066)	-0.092 (0.066)
Apps cause technical problems	0.165* (0.015)	0.169* (0.017)	0.162* (0.017)
Tools are easy to use	-0.191*** (<0.001)	-0.203*** (<0.001)	-0.195*** (<0.001)
Sum of ICT use for patient relationship	0.012 (0.571)	0.012 (0.588)	0.011 (0.588)
***Model statistics***			
N (observations)	423	423 (lower cens.: 11; upper cens.: 15)	423
R (multiple correlation)	0.530	—	—
R² (adjusted R²)	0.281 (0.266)	—	—
Log-likelihood	—	-525.72	-525.72
AIC/ BIC	1019.2/ 1059.7	1073.4/ 1118.0	1073.4/ 1118.0
F (p)	17.96 (<0.001)	—	—
σ (scale/ RMSE)	0.788	0.833	0.833
AME scaling factor	—	—	0.959

***Note.*** *Unstandardised coefficients (B); p-values in parentheses. OLS: ordinary least squares estimation on all 423 observations, exactly replicating the SPSS output. Tobit: doubly-censored Tobit regression in [1, 5]* estimated by maximum likelihood; β refers to the latent variable y*. OLS-Tobit: average marginal effects (AME) of the Tobit, ∂E[y|x]/∂x_j_ = β_j_· 1/N· Σ_i_ [Φ((U − X_i_β)/σ̂) − Φ((L − X_i_β)/σ̂)], directly comparable to OLS coefficients. *** p < 0.001; ** p < 0.01; * p < 0.05.

**Table 8:** Explanatory factors of techno-insecurity (TIN) in physicians.

Variable	OLS	Tobit	OLS-Tobit
	*Coef. (p)*	*Latent β (p)*	*AME (p)*
Constant	1.588*** (<0.001)	1.445*** (<0.001)	1.324*** (<0.001)
Age (years)	0.018*** (<0.001)	0.020*** (<0.001)	0.018*** (<0.001)
Women × Hospital care (interaction)	0.263*** (<0.001)	0.284*** (<0.001)	0.260*** (<0.001)
Women × Metropolitan area (interaction)	-0.191** (0.009)	-0.219** (0.006)	-0.201** (0.006)
Experience with telemedicine and digital health	-0.071 (0.092)	-0.081 (0.078)	-0.074 (0.078)
Some patients request it	0.085 (0.064)	0.093 (0.063)	0.085 (0.063)
Citizens easily access apps	-0.129** (0.003)	-0.128** (0.007)	-0.118** (0.007)
Apps cause technical problems	-0.023 (0.700)	-0.020 (0.757)	-0.018 (0.757)
Tools are easy to use	-0.065 (0.156)	-0.070 (0.161)	-0.064 (0.161)
Sum of ICT use for patient relationship	0.014 (0.422)	0.015 (0.434)	0.014 (0.434)
***Model statistics***			
N (observations)	423	423 (lower cens.: 42; upper cens.: 1)	423
R (multiple correlation)	0.356	—	—
R² (adjusted R²)	0.127 (0.108)	—	—
Log-likelihood	—	-481.60	-481.60
AIC/ BIC	903.1/ 943.6	985.2/ 1029.7	985.2/ 1029.7
F (p)	6.66 (<0.001)	—	—
σ (scale/ RMSE)	0.687	0.752	0.752
AME scaling factor	—	—	0.916

***Note.*** *Unstandardised coefficients (B); p-values in parentheses. OLS: ordinary least squares estimation on all 423 observations, exactly replicating the SPSS output. Tobit: doubly-censored Tobit regression in* [1, 5] estimated by maximum likelihood; β refers to the latent variable y*. OLS-Tobit: average marginal effects (AME) of the Tobit, ∂E[y|x]/∂x_j_ = β_j_· 1/N· Σ_i_ [Φ((U − X_i_β)/σ̂) − Φ((L − X_i_β)/σ̂)], directly comparable to OLS coefficients. *** p < 0.001; ** p < 0.01; * p < 0.05.

**Table 9:** Explanatory factors of techno-uncertainty in physicians.

Variable	OLS	Tobit	OLS-Tobit
	*Coef. (p)*	*Latent β (p)*	*AME (p)*
Constant	2.150*** (<0.001)	2.163*** (<0.001)	2.135*** (<0.001)
Age (years)	0.012*** (<0.001)	0.013*** (<0.001)	0.013*** (<0.001)
Women × Hospital care (interaction)	0.191* (0.016)	0.202* (0.011)	0.200* (0.011)
Women × Metropolitan area (interaction)	-0.159* (0.033)	-0.163* (0.029)	-0.161* (0.029)
Experience with telemedicine and digital health	-0.015 (0.731)	-0.017 (0.698)	-0.016 (0.698)
Some patients request it	0.064 (0.172)	0.064 (0.174)	0.063 (0.174)
Citizens easily access apps	-0.075 (0.092)	-0.077 (0.086)	-0.076 (0.086)
Apps cause technical problems	0.171** (0.004)	0.169** (0.005)	0.167** (0.005)
Tools are easy to use	-0.063 (0.176)	-0.068 (0.150)	-0.067 (0.150)
Sum of ICT use for patient relationship	0.040* (0.029)	0.040* (0.030)	0.039* (0.030)
***Model statistics***			
N (observations)	423	423 (lower cens.: 1; upper cens.: 7)	423
R (multiple correlation)	0.324	—	—
R² (adjusted R²)	0.105 (0.085)	—	—
Log-likelihood	—	-459.03	-459.03
AIC/ BIC	917.2/ 957.7	940.1/ 984.6	940.1/ 984.6
F (p)	5.37 (<0.001)	—	—
σ (scale/ RMSE)	0.699	0.711	0.711
AME scaling factor	—	—	0.987

***Note.*** *Unstandardised coefficients (B); p-values in parentheses. OLS: ordinary least squares estimation on all 423 observations, exactly replicating the SPSS output. Tobit: doubly-censored Tobit regression in [*1, 5] estimated by maximum likelihood; β refers to the latent variable y*. OLS-Tobit: average marginal effects (AME) of the Tobit, ∂E[y|x]/∂x_j_ *= β*_j_*· 1/N· Σ_i_ [Φ((U − X*_i_*β*)/*σ̂*) − *Φ*((L − X_i_*β*)/σ̂)], directly comparable to OLS coefficients. *** p < 0.001; ** p < 0.01; * p < 0.05.

Techno-insecurity is the empirical case where the analytical value of the doubly censored Tobit specification is most clearly demonstrated. With 42 lower-bound observations (10.2% of the sample), OLS estimates are systematically attenuated by approximately 8.4% relative to the latent Tobit β, with the strongest divergences observed in the most influential predictors of the dimension (Women × Hospital, Women × Metropolitan, age). The AME column recovers the OLS scale while preserving the censoring correction, providing the analytically most appropriate basis for policy quantification on the observed scale. By contrast, in techno-uncertainty (1.9% censoring), the three estimators are numerically equivalent and OLS can be reported with confidence [29,30]. The intermediate dimensions (TO at 4.5%, TI at 5.4%, TC at 6.1%) display modest but detectable divergences, with AME factors of 0.969, 0.943 and 0.959 respectively, that warrant the triangulation but do not substantively alter the inferences.

This pattern provides a methodological contribution to the medical-informatics literature on technostress measurement: the analytical relevance of the censoring correction is not uniform across dimensions, and the choice of estimator should be informed by the empirical censoring profile of each outcome rather than by a generic decision rule. The triangulation of OLS, Tobit and AME is the most parsimonious way to detect when the censoring correction matters and when it does not.

Three patterns emerge consistently across the five dimensions. First, the items capturing the type of digital engagement are the dominant family of predictors: at least one digital engagement item is significant in every dimension, and the perceived-ease-of-use item is significant in four of the five dimensions (all except techno-insecurity), confirming usability as a transversal driver of physician technostress, in line with the literature findings^30,32.^ Second, age operates as a significant determinant of three of the five dimensions (complexity, insecurity and uncertainty), with the strongest association in techno-complexity (b = +0.025 per year), supporting the cohort interpretation advanced in the global model. Third, the gender × context interactions display a coherent territorial structure across dimensions: Women × Hospital is positive and significant for techno-insecurity and techno-uncertainty (and approaches significance in techno-complexity), while Women × Metropolitan is negative and significant for techno-complexity, techno-insecurity and techno-uncertainty. The two interactions consistently operate in opposite directions, indicating that gender associations on physician technostress are not gender-uniform but concentrate in specific structural niches, exactly as predicted by the gendered EHR-burden literature [34].

Each technostressor also presents distinctive patterns that the multi-model analysis allows us to recover. Techno-overload is structurally tool-driven and digital-use-volume-driven: only the usability and reliability items, plus the volume of clinical ICT use, are significant. Techno-invasion is tool-design and patient-driven: ease of use, patient pull and citizen self-service are the only significant predictors, while no demographic or contextual variable matters. Techno-complexity is the dimension most fully captured by the model, with six significant predictors that include the strongest association of telemedicine experience (b = −0.309), confirming the mastery-through-experience mechanism. Techno-insecurity is the dimension most heavily driven by demographic and contextual variables (age, gender × context interactions) rather than by tool-level characteristics, indicating that the perceived threat to professional identity from digitalization is a sociodemographic rather than usability-driven phenomenon. Techno-uncertainty is dominated by technical-reliability problems and by the gender and context interactions, suggesting that the perception of technological instability is mediated by tool reliability and by structural niches.

## Discussion

A total of 423 physicians participated in the study, including professionals from both primary care and hospital settings. This study adopts an exploratory and explanatory approach, providing insight into the factors related to TS and its dimensions. The obtained results advance three analytical contributions to the empirical literature on technostress in healthcare. First, it triangulates three estimators: OLS, doubly censored Tobit and OLS-Tobit (AME) for an outcome that, although measured on a Likert scale, behaves empirically as a continuous variable in the observed range. The convergence of the three estimates provides robust evidence that the OLS specification preferred in the recent technostress literature [30,31] is not biased by the boundedness of the dependent variable when censoring shares are below 1%. In the same way, while preserving the option to switch to Tobit or AME when censoring is more pronounced. Second, the model decomposes the type-of-user construct into specific items capturing distinct facets of physicians’ digital engagement, revealing that perceived ease of use, accumulated telemedicine experience and patient demand-side pressure operate through partially distinct mechanisms. This decomposition is consistent with the structural-determinants framework increasingly adopted in digital Health [35] and provides a more granular set of intervention targets than aggregate adoption indices. Third, the model documents that gender effects on physician technostress are contextual (moderated by hospital-vs-other care setting and by metropolitan-vs-other location) rather than additive, an observation that has direct implications for the design of equity-oriented digital-transformation strategies in healthcare.

Regarding results, a notable finding is the high explanatory of age, that represents years of professional experience. Age and years of professional experience have also been described as relevant contributors to technostress among healthcare workers [41]. It is reasonable to assume that lower levels of digital literacy are associated with greater difficulty in using new technologies, and age has consistently been correlated with increased challenges in technology adoption [42]. In the current context, a substantial proportion of healthcare professionals in Catalonia are over the age of 55, which suggests that technostress may become a significant occupational health issue in the coming years. Moreover, in our sample the age association is robust to control for the sum of ICT uses in healthcare practice and for specific patterns of technology engagement. This result suggests that the underlying mechanism is generational digital skills rather than reduced exposure to or competence with specific digital tools. Therefore, the age outcomes reinforce the need to consider technostress not only as a user-level phenomenon, but also as a systemic issue embedded in workforce demographics and career trajectories. This interpretation aligns with the broader thesis advanced in literature that the digital determinants of health are socially patterned and stratify health-system actors along pre-existing inequality lines [35].

As in many other domains of health, a gender intersectional perspective also emerges in the experience of technostress. The use of ICT generates different levels of overall technostress and affects specific dimensions depending on physicians’ gender, their area of specialization and the territorial location of their workplace. Although gender differences in the use of new technologies and in their perceived usefulness in clinical practice have been described previously, their impact on overall technostress had not been documented until now. [43,44]. Higher technostress pathways observed among women hospital physicians is consistent with the gendered patterns of electronic health records-mediated workloads [45]. Protective association of metropolitan location among women physicians is consistent with the urban–rural digital-infrastructure gradient documented [35,36]: better connectivity, more mature deployment of digital-health systems and stronger IT-support resources in metropolitan settings reduce the friction that mediates technostress. The fact that gender does not enter the model as a stand-alone main effect but only through these two interactions empirically supports the methodological position of recent gender-and-digital-health scholarship: gender effects on physician technology experience are contextual, not additive.

Regarding digital technology uses, our results suggest a quantity effect: the more uses of digital technologies into medical practice, the greater the likelihood of technostress. In our baseline model, the association of clinical-ICT volume is statistically robust but quantitatively modest: it ranks seventh of the ten predictors in absolute standardized magnitude. The implication is that, conditional on the qualitative pattern of technology engagement, the marginal association of one additional ICT channel for patient interaction is real but small. This indicates that technostress does not arise from the volume of digital interaction per se, but rather from the nature and cognitive load of certain tasks. These findings challenge the widespread assumption that digital intensity is the primary driver of technostress and instead highlight the importance of examining task-specific burdens. Healthcare organisations attempting to mitigate technostress should therefore prioritize the qualitative dimensions of ICT use over absolute reduction of channel volume^40^.

Regarding interaction with technology, the research has identified five elements that influence physicians’ technostress. Among these, three protective factors were found: physicians’ experience with telemedicine and digital health, the perceived ease of use of the tools, and the ease of access citizens have to digital applications. However, two risk factors were also identified: patient demand for digital interaction and technical problems with digital applications.

Physicians who had previously engaged in digital health workflows reported significantly lower stress levels, likely due to greater digital confidence, increased familiarity with system logic, and reduced uncertainty [46]. This finding highlights the potential value of structured exposure, simulation-based training, and progressive implementation strategies to build digital resilience among clinicians [47]. On the other hand, it is important to highlight that one of the factors nuancing the highest levels of technostress among physicians is the perceived usefulness of digital applications and tools. Although there is solid evidence on the potential benefits of digital platforms, applications, and web-based resources [48], if clinicians do not perceive that a given tool improves clinical processes or enhances patient care, it ceases to be useful and may instead become a source of technostress. Although our data do not directly test non-linearity, one possible interpretation is that perceived usefulness may become protective only when accompanied by usability, reliability and adequate workflow integration. Conversely, digital tools that are perceived as useful or even necessary may still contribute to technostress if they increase cognitive load, fragment workflows or create dependency on systems that are not technically reliable. This interpretation should therefore be considered exploratory and should be examined in future studies using designs able to test non-linear associations between perceived usefulness and technostress.

At very low levels of perceived usefulness, professionals may experience insecurity or disengagement; at very high levels, increased dependency and workload may lead to overload. Only when usefulness is matched with sufficient usability, reliability, and adequate workflow integration becomes a protective factor. This nuance is important for interpreting technostress in real clinical environments, where digital tools that are perceived as indispensable may also intensify cognitive demands. Similarly, citizens’ accessibility and reliability play a critical role. Any digital application that fails to function properly from the outset can shape clinicians’ perceptions permanently. Ensuring that digital tools work seamlessly is therefore essential, as early negative experiences can heavily influence acceptance. Conversely, when professionals find digital tools useful, their increased use of these technologies becomes a protective factor against technostress.

Social influence also showed a significant association with overall technostress and their dimensions, indicating that expectations or behaviors from citizens and patients can amplify stress levels. Patients influence may operate through subtle mechanisms of normative pressure, competition, or perceived social obligation to adopt digital tools that others find useful. These dynamics underline the importance of organisational culture in shaping digital wellbeing, as peer-driven implementation can inadvertently widen gaps between early adopters and those who struggle with technology.

Technical difficulties emerged as consistent predictors of all technostress dimensions in our models. This finding underscores the central role of system reliability in shaping physicians’ perceptions of digital tools. When digital applications malfunction, respond slowly, or present usability issues, they not only hinder workflow but also erode trust in the entire digital ecosystem. Our results align with prior evidence showing that system quality is a key determinant of technology acceptance and psychological strain. In this sense, addressing technical problems is likely one of the most effective strategies to reduce technostress, as even highly useful or well-designed tools become stress-inducing if they fail to operate consistently. These roles aim to improve patients’ engagement and adherence to digital tools, ultimately reducing the burden on healthcare professionals. By enhancing digital inclusion and supporting users in navigating technological systems, digital facilitators may become a key strategy not only for improving population-level access but also for mitigating technostress among clinicians [49].

Importantly, the joint inclusion of these five items reveals the multidimensional nature of the type-of-user construct. Aggregating these items into a single index (as is common in earlier technostress research) would obscure the fact that the construct contains both protective and risk-conferring components. The item-level decomposition is analytically necessary to recover this structure and produces directly actionable design and governance implications^40^.

Two important organizational routines emerge from the findings in this category of predictors. First, because of the direct pressure users exert on physicians, the importance of organizational protocols that absorb patient-initiated digital demand, rather than transferring it directly to healthcare professionals, is highlighted. A second organizational implication arising from the stress caused by technical problems (beyond usability) is that physicians distinguish between intrinsic usability and the operational reliability of digitals tools. This distinction is relevant for digital health management, that must consider both issues.

From a health policy perspective, the empirical results support a focused intervention agenda. Investments in digitals tools usability and in structured, longitudinal exposure of physicians to telemedicine workflows are may generate the largest reductions in technostress per unit of organisational effort. Conversely, simply reducing the volume of patient-relationship ICT channels is a less efficient strategy than improving the qualitative dimensions of those channels. The persistent age association, robust to all controls, suggests that targeted re-skilling programmes for the older cohorts of physicians are a complementary necessary lever. Finally, the gender-by-context interactions suggest that the digital-transformation gap is not gender-uniform but concentrates in specific structural niches: women in hospital settings outside metropolitan areas. Equity-oriented digitalization policies should therefore identify and prioritize these niches, rather than rely on universal interventions.

## Limitations

This study contributes to the emerging analysis of technostress in physicians by examining associations between individual, professional and contextual characteristics and technostress. However, several limitations should be explicitly considered when interpreting the findings. First, the study used a non-probabilistic convenience sample of physicians recruited through professional channels, email dissemination and social media. Consequently, the sample cannot be assumed to be representative of all physicians in Catalonia or other healthcare systems. In addition, because the total number of professionals who received, opened or were exposed to the survey invitation could not be determined, a conventional response rate could not be calculated and non-response bias cannot be ruled out. Therefore, the descriptive estimates should not be interpreted as population prevalence estimates.

Second, the cross-sectional design precludes causal inference. The associations observed between technostress and sociodemographic, professional and technological variables should be interpreted as conditional associations rather than causal effects. Although the regression models identify factors statistically associated with higher or lower technostress, they cannot establish temporality or determine whether these factors cause technostress. Longitudinal or experimental studies are needed to assess causal pathways and to evaluate whether interventions targeting usability, technical reliability or digital training reduce technostress over time.

Third, technostress was assessed using self-reported measures, which may introduce biases stemming from subjective perceptions, social desirability or common method variance. Although common method bias was formally assessed, residual measurement bias cannot be fully excluded. Fourth, the overall technostress R² of 0.21 indicates that meaningful variance remains unexplained, as expected for an outcome partly driven by individual-level psychosocial factors not included in the present specification. Fifth, the single-region sample limits external validity, although the substantive patterns documented in the models are coherent with previous evidence from other healthcare contexts.

Finally, the study did not specifically explore the impact of more recent digital technologies, such as artificial intelligence, surgical robotics or virtual reality, which may generate different sources of technology-related strain in healthcare work. Future studies should investigate how emerging tools, including AI-powered systems, affect different professional healthcare groups.

## Conclusion

Taken together, our findings reveal that technostress among physicians is shaped by a complex interplay of sociodemographic, professional and technological factors. Technostress is more likely to emerge in specific groups of professional and tasks: older physicians, women working in hospitals, and those in non-metropolitan areas, where technology is more prevalent in-patient care, patients also have access to and pressure for technology use, and where technical problems are common. However, research has also identified three factors that moderate technostress: physicians’ experience with eHealth and telemedicine applications, greater usability of digital tools, and improved public access. Addressing these intersectional domains in an integrated manner will be essential for promoting digital wellbeing of physicians and ensuring a sustainable digital transformation of healthcare systems.

## Supporting information

S1 Data Database of the professionals who have participated in the study. (SAV)
