# Supplementary material for: Beyond technology usefulness: Health system reliability, and socio-demographic and professional factors as shapers of technostress in physicians
Source: PLOS Digit Health. 2026 Jul 30;5(7):e0001541. doi: 10.1371/journal.pdig.0001541 (PMC13423038; doi:10.1371/journal.pdig.0001541)
Supplement: Attachment — Submitted filename: Response_to_reviewers_PLOSDH(1).docx [file pdig.0001541.s003.docx]

**Answer to the review on the paper**

**“Beyond technology usefulness: Health system reliability, and socio-demographic and**

**professional factors as shapers of technostress in physicians” (PDIG-D-26-00165)**

**General remarks**

**Authors comment:**

Thank you for inviting us to resubmit Manuscript ID Ref. PDIG-D-26-00165, entitled "Beyond technology usefulness: Health system reliability, and socio-demographic and professional factors as shapers of technostress in physicians " for possible publication in PLOS Digital Health.

We sincerely appreciate your feedback and the thoughtful comments provided by the reviewers, which have helped us improve the manuscript. In response, we have made substantial revisions, which are highlighted in yellow (attending the responses to Reviewer 1 and Reviewer 2) throughout the revised-tracked version.

Below is a summary of the key changes made:

- Following the recommendations of both reviewers, we have addressed the robustness issues in the article's empirical approach (regressions). Specifically, we propose a completely new regression strategy, estimating three different models (OLS, Tobit, and Tobit-OLS). This has entailed a complete rewriting of this section of the article.
- In this regard, the presentation of results (tables and text) has been redesigned to better connect the different sections of the article.
- In this new version of the article, we go beyond the purely technological antecedents of technostress and begin to unravel an initial set of sociodemographic and professional factors that drive technostress among physicians. We have been able to identify specific groups of physicians and the relevance of experience, which, together with professional and usability factors, are antecedents of technostress. In this way, we recommend intersectional interventions to prevent technostress and the entire range of occupational health and well-being problems that can be associated with it.

We are grateful for the opportunity to revise and resubmit our work and hope that the revised manuscript meets the expectations of the reviewers and the journal.

***Detailed answer to reviewer’s comments and recommendations***

| **Reviewer’s comment** | **Author’s response** |
| --- | --- |
|  |  |
| **Reviewer 1 (changes and additions to the manuscript, marked in yellow)** | |
| 1. The major problem of this text is the insufficient representativeness of the sample, and the sampling method cannot calculate the response rate. | We thank the reviewer for raising the issue of sample representativeness. We acknowledge that the study uses an incidental (convenience) sample of 423 physicians in Catalonia and that a conventional response rate cannot be computed: the questionnaire was disseminated through professional channels (medical college mailings, professional society newsletters and physician social-media networks) for which the true denominator of exposure is undefined, a structural feature of contemporary online survey research with health professionals rather than a unique limitation of the present study.  However, we estimate that the survey was potentially accessible to over 2000 physicians in Catalonia through professional associations and social media platforms. This limitation precluded calculation of a precise response rate but highlights the need for caution when interpreting the representativeness of the sample. We submit that this design is nevertheless appropriate for the inferential aims of the manuscript on three grounds.  First, the study is not designed to estimate the population prevalence of technostress (which would indeed require probability sampling and a known sampling frame); it is designed to estimate the associations between sociodemographic, organisational and technology-use variables on the one hand, and self-reported technostress on the other. The bias introduced by non-probability sampling on regression coefficients is known to be substantially smaller than on descriptive parameters, because the relevant selection mechanisms are largely captured by the covariates included in the model (Cornesse et al. 2020).  Second, the achieved sample size (N = 423) yields a case-to-predictor ratio of 42:1 for the ten-parameter specification reported in Table 1, comfortably above the 10:1 to 20:1 thresholds conventionally recommended for OLS inference (Hair et al. 2019).  Third, the principal threat to validity in cross-sectional self-report designs of this type (i.e. common method bias) has been formally tested and ruled out using Harman's single-factor procedure (Podsakoff et al. 2003), with the first unrotated component accounting for only 16.9% of the variance, well below the 50% threshold.  Furthermore, incidental and convenience sampling is the dominant practice in the empirical literature on technostress in healthcare professionals. Recent scoping reviews documents that the vast majority of published studies in this field rely on non-probability samples drawn from single institutions, professional networks or online dissemination. This is the case for the most cited contributions, including Tajirian et al. (2020), Golz et al. (2021), Califf et al. (2020) or Rotenstein et al. (2024).  Our design therefore conforms to standard practice in the field, while incorporating a **representativeness and explicit common-method-bias diagnostic** that not all these prior studies report. Also, the **Limitations** section of the revised manuscript states this explicitly and invites future work to triangulate our findings on probability-based samples where feasible.  References:   - Cornesse C, Blom AG, Dutwin D, Krosnick JA, De Leeuw ED, Legleye S, et al. A review of conceptual approaches and empirical evidence on probability and nonprobability sample survey research. *Journal of Survey Statistics and Methodology*. 8.1 (2020):4–36. - Cunningham CT, Quan H, Hemmelgarn B, Noseworthy T, Beck CA, Dixon E, et al. Exploring physician specialist response rates to web-based surveys. *BMC Medical Research Methodology*. 2015;15:32. - Golz C, Peter KA, Müller TJ, Mutschler J, Zwakhalen SMG, Hahn S. Technostress and digital competence among health professionals in Swiss psychiatric hospitals: cross-sectional study. *JMIR Mental Health*. 2021;8(11):e31408. - Tajirian T, Stergiopoulos V, Strudwick G, Sequeira L, Sanches M, Kemp J, et al. The influence of electronic health record use on physician burnout: cross-sectional survey. *Journal of Medical Internet Research*. 2020;22(7):e19274 - Califf CB, Sarker S, Sarker S. The bright and dark sides of technostress: a mixed-methods study involving healthcare IT. *MIS Quarterly*. 2020;44(2):809–856 - Rotenstein LS, Hendrix N, Phillips RL, Adler-Milstein J. Team and electronic health record features and burnout among family physicians. *JAMA Network Open.* 2024;7(11):e2443857. |
| 2. The adjustments of all regression models exceed 0.90, which is extremely rare in behavioral and health research and should be interpreted with caution. | We are grateful to the reviewer for this important observation regarding the unusually high R² values. On re-examination of the original specification, we acknowledge that the no-intercept models reported uncentred R² values (1 − SSR / Σy²) which, for a Likert outcome with strictly positive support, are mechanically larger than the conventional centred R². Combined with the decision to suppress the constant to facilitate the reading of coefficients as net contributions, this generated R² magnitudes that may be misleading. In the revised manuscript we have therefore restructured the analytical strategy as follows. First, we restore OLS with intercept as the reference specification, which yields adjusted R² = 0.193 — well within the range typical of the technostress literature on healthcare professionals (Bail et al. 2023; Golz et al. 2021). Second, we add a doubly censored Tobit model in [1, 5] that explicitly accounts for the bounded nature of the Likert outcome and corrects, by maximum likelihood, the attenuation that OLS would suffer in the presence of floor or ceiling effects (Austin et al. 2000). Third, we report the OLS-Tobit average marginal effects (AME), which translate the latent Tobit β coefficients back onto the observed scale of OLS by multiplying them by the average probability that an observation lies strictly within the censoring bounds, providing a single set of coefficients that is simultaneously OLS-comparable and censoring-corrected.  The triangulation across the three estimators (**Table 4**) shows that, with a censoring share of 0.24% and an AME scaling factor of 0.999, OLS, Tobit β and AME estimates are practically indistinguishable to decimal places, confirming (rather than by assumption) that the substantive inferences are robust to the choice of estimator and that the OLS specification is not biased by the boundedness of the dependent variable in this sample.  The coefficient results regarding the role of age, the gender × care setting and gender × territory interactions, eHealth ant telemedicine experience, the perceived ease of use, patient digital health demand, technical problems, ICT volume in patient care are stable across the three specifications, supporting the substantive solidity of the findings independently of the econometric framework adopted.  This new empirical strategy has led to a complete rewriting of the manuscript section: **Associated factors to technostress and technostressors**. In addition, a new version of the tables on the antecedents of technostress and its technostressors is presented (**Tables 4 to 9**).  **References:**  Austin PC, Escobar M, Kopec JA. The use of the Tobit model for analyzing measures of health status. Quality of Life Research. 2000;9(8):901–910.  Bail C, Harth V, Mache S. Digitalization in urology — A multimethod study of the relationships between physicians' technostress, burnout, work engagement and job satisfaction. Healthcare. 2023;11(16):2255.  Golz C, Peter KA, Müller TJ, Mutschler J, Zwakhalen SMG, Hahn S. Technostress and digital competence among health professionals in Swiss psychiatric hospitals: cross-sectional study. JMIR Mental Health. 2021;8(11):e31408. |
| 3. The information in the table is redundant and the layout is chaotic. | Thank you very much for your feedback. After completely redesigning the empirical strategy, we have entirely revised the regression results tables (**Tables 4 to 9)**. To improve their informational value, each table now presents only the regression results for the three specified models (OLS, Tobit, OLS-Tobit-AME) and their main robustness statistics. |
| 4. The author needs to check the consistency between the result text and the table data item by item, especially in terms of coefficient size and significance level. | We thank the reviewer for this important observation. The section **Associated factors to technostress and technostressors** is entirely new. In addition to presenting the new results, we have paid particular attention to the content of the coefficients (and their correspondence with the tables presented), the organization, and the narrative explaining the results. |
| 5. The author uses causal words, but the study is a cross-sectional design and cannot infer causal relationships. | Thank you again for your comment. In explaining the regression results, we removed our claims about causality and restricted the associations found between explanatory and explained variables in terms of predictive capacity or antecedents of technostress. (see the section **Associated factors to technostress and technostressors**). |
| 6. The author proposed to integrate structural, perceptual and situational factors, but did not provide a targeted intervention direction. | Another strong argument for improving our article. The article's strategy has been precisely to go beyond its purely technological antecedents and begin to unravel an initial set of sociodemographic and professional factors that drive technostress among physicians. With this first approach, we have been able to identify specific medical groups and the relevance of experience, which, together with usability factors, are antecedents of technostress. In this way, healthcare institutions and authorities have new evidence to implement policies and practices to prevent technostress and the entire range of occupational health and well-being problems that can be associated with it. Based on the results obtained, the **discussion and conclusion sections** propose some specific intersectional intervention strategies. |
|  |  |
| **Reviewer 2 (changes and additions to the manuscript, marked in yellow)** | |
| This manuscript addresses a relevant and timely topic in digital health, centred on the increasing burden of technostress among physicians as healthcare systems undergo rapid digital transformation. The focus on moving beyond simple measures of technology use toward a broader consideration of system reliability, contextual influences, and professional characteristics is particularly valuable and supports a more comprehensive, multidimensional understanding of technostress. The use of the Technostress Creators Scale, together with the inclusion of both individual (sociodemographic and experiential) and organisational/contextual variables, provides a solid empirical foundation for the analysis. | Thank you very much. Our approach, and we believe one of the main contributions of this article, is to expand upon traditional descriptive analyses of technostress in healthcare professionals. Going beyond typical models of technology acceptance and use, we analyze the predictive effects that the technological reliability of the healthcare system, and the sociodemographic and professional characteristics of physicians, have on their perception of technostress. To this end, we analyzed a convenience sample of 423 physicians from the healthcare system in Catalonia (Spain). |
| The manuscript is generally well structured and clearly written, although some sections would benefit from minor clarification and tighter alignment between results and interpretation. The research questions are well aligned with the analytical strategy, and the sample size is adequate for exploratory analysis. The emphasis on technical problems and accessibility as key determinants of technostress is especially compelling and has clear practical implications for healthcare organisations. Likewise, it offers an important nuance that challenges the assumption that digital intensity is the primary driver of technostress. | Thank you very much for your comments. The article's strategy has been precisely to go beyond its purely technological antecedents and begin to unravel an initial set of sociodemographic and professional factors that drive technostress among physicians. With this first approach, we have been able to identify specific medical groups and the relevance of experience, which, together with usability factors, are antecedents of technostress. In this way, healthcare institutions and authorities have new evidence to implement policies and practices to prevent technostress and the entire range of occupational health and well-being problems that can be associated with it. |
| However, there are several aspects of the analysis and interpretation that would benefit from further clarification and, in some cases, reconsideration. The most important issue concerns the very high proportion of variance explained by the regression models, particularly in the first step where gender and years in practice alone appear to account for the vast majority of technostress variance. Given that these two variables appear to explain over 90% of the variance, this result is highly unusual in psychosocial research and warrants closer scrutiny. While the authors themselves note that this is unusually high, the implications of this finding are not fully explored. In its current form, the result raises questions about model specification, potential overlap between predictors and outcome measures, or other sources of statistical inflation. It would be helpful for the authors to provide additional diagnostic information and a more detailed explanation of how these results should be interpreted. | We are grateful to the reviewer for this important observation regarding the unusually high R² values. On re-examination of the original specification, we acknowledge that the no-intercept models reported uncentred R² values (1 − SSR / Σy²) which, for a Likert outcome with strictly positive support, are mechanically larger than the conventional centred R². Combined with the decision to suppress the constant to facilitate the reading of coefficients as net contributions, this generated R² magnitudes that may be misleading. In the revised manuscript we have therefore restructured the analytical strategy as follows. First, we restore OLS with intercept as the reference specification, which yields adjusted R² = 0.193 — well within the range typical of the technostress literature on healthcare professionals (Bail et al. 2023; Golz et al. 2021). Second, we add a doubly censored Tobit model in [1, 5] that explicitly accounts for the bounded nature of the Likert outcome and corrects, by maximum likelihood, the attenuation that OLS would suffer in the presence of floor or ceiling effects (Austin et al. 2000). Third, we report the OLS-Tobit average marginal effects (AME), which translate the latent Tobit β coefficients back onto the observed scale of OLS by multiplying them by the average probability that an observation lies strictly within the censoring bounds, providing a single set of coefficients that is simultaneously OLS-comparable and censoring-corrected.  The triangulation across the three estimators (**Table 4**) shows that, with a censoring share of 0.24% and an AME scaling factor of 0.999, OLS, Tobit β and AME estimates are practically indistinguishable to decimal places, confirming (rather than by assumption) that the substantive inferences are robust to the choice of estimator and that the OLS specification is not biased by the boundedness of the dependent variable in this sample.  The coefficient results regarding the role of age, the gender × care setting and gender × territory interactions, eHealth ant telemedicine experience, the perceived ease of use, patient digital health demand, technical problems, ICT volume in patient care are stable across the three specifications, supporting the substantive solidity of the findings independently of the econometric framework adopted.  This new empirical strategy has led to a complete rewriting of the manuscript section: **Associated factors to technostress and technostressors**. In addition, a new version of the tables on the antecedents of technostress and its technostressors is presented (**Tables 4 to 9**).  **References:**  Austin PC, Escobar M, Kopec JA. The use of the Tobit model for analyzing measures of health status. Quality of Life Research. 2000;9(8):901–910.  Bail C, Harth V, Mache S. Digitalization in urology — A multimethod study of the relationships between physicians' technostress, burnout, work engagement and job satisfaction. Healthcare. 2023;11(16):2255.  Golz C, Peter KA, Müller TJ, Mutschler J, Zwakhalen SMG, Hahn S. Technostress and digital competence among health professionals in Swiss psychiatric hospitals: cross-sectional study. JMIR Mental Health. 2021;8(11):e31408. |
| Relatedly, some aspects of the statistical reporting would benefit from clarification. The discussion of multicollinearity appears to contain a likely inconsistency in the interpretation of tolerance and VIF thresholds, as the stated criteria (tolerance < 0.10 and VIF > 10) would typically indicate problematic multicollinearity rather than its absence. In addition, the use of ordinary least squares regression alongside non-parametric correlation analyses could be more explicitly justified, particularly given the combination of non-parametric correlations with OLS regression (albeit with bootstrapped estimates). Providing a clearer rationale for these analytical choices, along with additional information on model assumptions and diagnostics, would strengthen the methodological section of the paper. | We thank the reviewer for this methodological observation. Regarding multicollinearity thresholds, we acknowledge that the original wording inverted the conventional interpretation of the diagnostics and have corrected it in the revised manuscript. The standard criterion is that tolerance values above 0.10 and VIF values below 10 indicate the absence of problematic multicollinearity. For the new OLS specification (N = 423, ten parameters), the maximum observed VIF is 1.23 (item patient request) and the minimum tolerance is 0.81, well within even the strict thresholds (VIF < 5 or VIF < 2.5) recommended for models with interaction terms. The two interactions (Women × Hospital, Women × Metropolitan) display VIFs of 1.18 and 1.12 respectively, confirming that the orthogonalisation between gender, care setting and territory in the sample is sufficient to avoid the non-essential collinearity that interaction terms can mechanically generate. The Belsley-Kuh-Welsch maximum condition index is 28.30, below the conventional cut-off of 30.  As in the OLS specification, in the specified Tobit and OLS-Tobit models, multicollinearity tests also rule out its presence. A paragraph explaining the treatment of multicollinearity has been added to the section **Associated factors to technostress and technostressors**. |
| From a conceptual perspective, the interpretation of perceived usefulness as a positive predictor of technostress is intriguing but somewhat counterintuitive in light of established models such as TAM and UTAUT. The authors offer an interesting speculative explanation in terms of a possible non-linear relationship, but this interpretation would benefit from being framed more cautiously unless directly tested. It would also be helpful to more explicitly reconcile this finding with the negative bivariate associations reported earlier in the manuscript. As it stands, the argument is thought-provoking and could represent an important contribution, but it would be helpful to distinguish more clearly between empirical findings and theoretical interpretation. | We thank the reviewer for these conceptual observations, which have led to a substantial improvement in both the **results and the discussion sections** of the article. We address them in two (related) parts.   1. Resolution of the original counterintuitive finding. In the revised specification (Table 1), the technology-use block has been decomposed into theoretically distinct sub-dimensions, separating perceived ease of use (a TAM construct) from the volume of digital channels deployed in patient interactions (Sum_ICT uses for patient relationship, a job-demands construct). Perceived ease of use now carries the negative coefficient predicted by TAM/UTAUT (β = −0.150, p < 0.001), while ICT volume carries the positive coefficient predicted by the techno-overload literature (β = +0.033, p = 0.018). The previously counterintuitive coefficient on perceived usefulness for professional development is no longer in the model; we note in passing that this dropped item shows a negative bivariate Spearman correlation with technostress (ρ = −0.146, p = 0.003), confirming that the original anomalous sign was a specification artefact rather than a substantive reversal of the TAM mechanism. 2. Bivariate–multivariate reconciliation. Every predictor retained in the multivariate model shows the same sign as its bivariate Spearman correlation with technostress (Tools are easy to use: ρ = −0.277, β = −0.150; Telemedicine & eHealth experience: ρ = −0.129, β = −0.086; Citizens easily access apps: ρ = −0.173, β = −0.101; Apps cause technical problems: ρ = +0.302, β = +0.109; Sum_ICT uses for patient relationship: ρ = +0.088, β = +0.033). Therefore, the bivariate and multivariate evidence are therefore mutually consistent. |
| Overall, this is a thoughtful and well-developed manuscript that addresses an important topic with clear relevance for both research and practice. The authors bring a strong understanding of the field, and the study has the potential to make a meaningful contribution to the literature on digital health and clinician wellbeing. With some further clarification of the statistical findings and a slightly more cautious interpretation of certain results, the paper would be significantly strengthened and well positioned for publication. | Thank you very much for this positive assessment and especially for the comments and suggestions for improvement. We believe that with the new empirical regression strategy and the improved specification of working hypotheses, results, and implications, the article has been strengthened. |
|  |  |
