# Supplementary material for: Beyond technology usefulness: Health system reliability, and socio-demographic and professional factors as shapers of technostress in physicians
Source: PLOS Digit Health. 2026 Jul 30;5(7):e0001541. doi: 10.1371/journal.pdig.0001541 (PMC13423038; doi:10.1371/journal.pdig.0001541)
Supplement: Attachment — Submitted filename: Reviewe Response.docx [file pdig.0001541.s004.docx]

Dear Editor,

We thank the reviewer for this important observation. We have revised the Abstract and Limitations sections to state more explicitly that the study used a non-probabilistic convenience sample and that a conventional response rate could not be calculated. We now also clarify that the cross-sectional design precludes causal inference and that the results should be interpreted as exploratory conditional associations rather than causal effects or population prevalence estimates. Accordingly, we have softened causal wording throughout these sections and have indicated that longitudinal or experimental studies are needed to assess causal pathways.

Dr. Oriol Yuguero Torres
